# A new genome assembly of the pea cultivar ‘Caméor’ provides resources for functional genomics and genetics

**DOI:** 10.1038/s41597-026-07347-4

**Published:** 2026-05-12

**Authors:** Jonathan Kreplak, Petr Novák, Laura Ávila Robledillo, Grégoire Aubert, Baptiste Imbert, Parwinder Kaur, Quentin Gouil, Céline Lopez-Roques, Nathalie Rodde, Olivier Bouchez, Nadim Tayeh, Jiří Macas, Judith Burstin

**Affiliations:** 1 Université Bourgogne Europe, Institut Agro Dijon, INRAE, UMR Agroécologie, Dijon, France; 2 Biology Centre, Czech Academy of Sciences, Ceske Budejovice, Czech Republic; 3 The University of Western Australia, 35 Stirling Highway, 6009 Perth, Australia; 4 Epigenetics and Development Division, The Walter and Eliza Hall Institute of Medical Research, Parkville, Victoria Australia; 5 Department of Medical Biology, The University of Melbourne, Melbourne, Victoria Australia; 6 Olivia Newton-John Cancer Research Institute, Heidelberg, VIC 3084 Australia; 7 School of Cancer Medicine, La Trobe University, Bundoora, VIC 3086 Australia; 8 INRAE, GeT-PlaGe, Genotoul, 31326 Castanet-Tolosan France; 9 INRAE CNRGV, 24 Chemin de Borde Rouge, Auzeville, CS 52627, 31326 Castanet Tolosan, France

**Keywords:** Plant sciences, Genomics

## Abstract

Significant improvements in sequencing technologies have allowed the development of more contiguous genome assemblies in many plant species. The pea genome is characterized by its richness in repeated elements and its long and complex centromeres. This makes its assembly challenging. In this paper, we present an improved version of the genome sequence of the French cultivar ‘Caméor’. This sequence was obtained by combining Nanopore and PacBio long-read sequencing, Hi-C contact maps and Bionano maps. The assembly of centromeres was refined using a combination of FISH and ultra-long Nanopore read analyses. Overall, the new Cameor_v2 genome assembly is a highly continuous pea genome assembly with small total gap size and a large contig N50. In this version, the orientation of chromosomes was revised according to internationally accepted karyotype rules. Gene annotation statistics indicated a high completeness of gene sequences, with most gene sequences with 3′ and 5′ UTR. This genome assembly with its associated data constitute a useful resource for pea genetics, comparative mapping and functional genomics.

## Background & Summary

Pea (*Pisum sativum* L., 2n = 14) is one of the most cultivated grain legumes in the world with soybean, common bean and chickpea^1^. Pea seeds, consumed both fresh (~21 M tons produced worldwide in 2022) and dry (14 M tons in 2022), are an essential source of proteins, fibres and micronutrients for human and animal nutrition. Moreover, the pea crop is an important component of agroecological cool-season cropping systems. Pea plants can acquire nitrogen through a symbiosis with N-fixing soil bacteria, reducing the need for synthetic fertilizers in crop rotations, and thus limiting greenhouse gas emission.

Pea breeding has long been hampered by the lack of a reference genome. The first draft assembly of the seven pea chromosomes was achieved for the French dry pea cultivar ‘Caméor’ using short-read sequencing in combination with physical and genetic mapping^2^. A significantly more contiguous assembly was produced for the Chinese variety ‘Zhongwan 6’ (ZW6)^3^ by using PacBio long-read sequencing. More recently, new pea genome assemblies were obtained for a vegetable Chinese pea variety, ‘Zhewan 1’ (ZW1)^4^, and the dwarf, wrinkled-seeds accession JI2822^5^.

The pea genome is rich in repetitive DNA consisting mainly of a diverse population of giant Ty3/gypsy Ogre elements^6^. In addition, pea chromosomes are characterized by highly elongated centromeres containing several separate domains of CENH3 chromatin (“meta-polycentromeres”^7^). There are twelve families of satellite DNA associated with pea centromeric chromatin^8^, some of which form arrays of nearly identical monomers that are up to 2 Mb in size. These features make the full assembly of the pea genome, and especially its centromeres, a particularly challenging task. However, the use of ultra-long Oxford nanopore and PacBio HiFi reads in combination with cytogenetic mapping has recently led to a nearly complete assembly of the 177.6 Mb region of pea chromosome 6, including its 81.6 Mb centromere (CEN6), thus demonstrating the feasibility of this approach for assembling the complete pea genome^9^.

In the present study, we aimed to generate a high-quality chromosome-level assembly of *P. sativum* cv. ‘Caméor’ by combining newly generated PacBio HiFi and ultra-long Oxford Nanopore reads with Hi-C and optical mapping data. The centromeres of the remaining six pea chromosomes were integrated into chromosome-level pseudomolecules using the approaches developed for CEN6 assembly and characterized using CENH3 ChIP-seq data. This new genome assembly, with carefully annotated genes and repetitive DNA, constitutes a high-quality reference genome for pea. Gene expression and SNP data were linked to it in order to provide a comprehensive resource for research and breeding.

## Methods

### Genomic DNA sequencing

High molecular weight (HMW) DNA was prepared from the nuclei extracted from leaf tissues of pea cv. ‘Caméor’ seedlings, as described previously^10^. DNA quality was checked using field inversion gel electrophoresis (FIGE) to ensure that the DNA fragment size was >100 kb. Whole genome sequencing included 73.1 Gb PacBio HiFi Sequel II reads and 119.6 Gb ultra-long Nanopore reads ranging from 30 to 801 kb in length (N50 = 83.8 kb) that were previously generated as described in Macas *et al*.^9^. Briefly, high molecular weight DNA was prepared from the nuclei extracted from young leaves. The quality of DNA preparations was checked using field inversion gel electrophoresis (FIGE) to ensure that the DNA fragment size was >100 kb. Then, 20 nanopore MinION (Oxford Nanopore Technologies) sequencing runs were done using 4 libraries prepared according to the manufacturer’s instructions (Table 1): SQK-LSK109 (library 1; 13 runs), SQK-LSK110 (library 2; 1 run), SQK-RAD004 (library 3; 3 runs), and SQK-ULK001 (library 4; 3 runs). Raw nanopore reads were basecalled using Oxford Nanopore basecaller Guppy (ver. 3.6.0 and 4.5.4). Raw sequence data are available from the ENA European Nucleotide Archive at https://identifiers.org/ena.embl:PRJEB54858^11^ under accession numbers ERR9972527-ERR9972530 (PacBio HiFi), ERR9980778-ERR9980797 (Oxford Nanopore).

**Table 1:** Summary of Oxford Nanopore sequencing data used for the Cameor V2 genome assembly.

Centre	Library	Flow Cell	Output (Gb)	N50 (kb)	Ref
BC CAS	1–4	MinIon	119.6	83.8*	Macas *et al*.^9^
Get-Plage	5	GridION 1	25	18.5	Present study
Get-Plage	5	GridION 2	25	18.5	Present study
Get-Plage	5	PromethION 1	31	15	Present study
Get-Plage	6	PromethION 2	52.9	17	Present study
Get-Plage	6	Flongle 1	1.1	19	Present study
Get-Plage	7	Flongle 2	0,5	22	Present study
Get-Plage	7	PromethION 3	91	22	Present study
Get-Plage	8	GridION 3	5.5	20	Present study
Get-Plage	8	PromethION 4	17.9	18	Present study
WEHI	9	PromethION	39	16	Present study
WEHI	10	PromethION	57	16	Present study

*Reads shorter than 30 kb were discarded.

Additional Nanopore reads were obtained from leaf genomic DNA isolated using Qiagen Genomic-tips 100/G kit (QIAGEN, Hilden, Germany) from two partners (Table 1).

At GeT-PlaGe (INRAE Toulouse), library preparation and sequencing were performed according to the manufacturer’s instructions “1D gDNA selecting for long reads (SQK-LSK109)”. At each step, DNA was quantified using the Qubit dsDNA HS Assay Kit (Life Technologies). DNA purity was tested using the NanoDrop (Thermofisher) and size distribution and degradation assessed using the Fragment analyzer (Agilent) DNF-464 HS Large Fragment Kit. Purification steps were performed using AMPure XP beads (Beckman Coulter). Three protocols were sequentially used for library preparation: Protocol 1: For libraries 5 and 6, 10 µg of DNA1 were purified then sheared at 40 kb using the Megaruptor 1 system (diagenode). A size selection step using Short Read Eliminator XS Kit (Circulomics) was performed. Using SQK-LSK 109 kit (ONT) libraries were loaded onto R.9.4.1 flowcells (9 fmol on 1 Flongle, 15 fmol on 2 GridION and 15 fmol and 18 fmol on 2 PromethION). Protocol 2: For library 7, 5 µg of DNA1 were purified then an extra repair step with SMRTbell DAMAGE REPAIR KIT (PACBIO, 100-465-900) was performed. A size selection step using Short Read Eliminator XS Kit (Circulomics) was performed. Using SQK-LSK 109 kit (ONT) library was loaded onto R.9.4.1 flowcells (8 fmol on 1 Flongle and 15 fmol on 1 PromethION). Protocol 3: For library 8, 20 µg of DNA2 were purified then an extra repair step with SMRTbell DAMAGE REPAIR KIT (PACBIO, 100-465-900) was performed. A size selection step using Short Read Eliminator XL Kit (Circulomics) was performed. Using SQK-LSK 109 kit (ONT) libraries were loaded onto R.9.4.1 flowcells (15 fmol on 1 GridION and 15 fmol on 1 PromethION). High accuracy live base calling was done with Guppy 3.2+.

In parallel, at Walter and Eliza Hall Institute of Medical Research, two libraries (library 9 and library 10) were prepared, each using 2 µg high-molecular weight genomic DNA resuspended in 48 µl water, with the SQK-LSK109 kit according to the manufacturer’s instructions except for extended FFPE/End repair incubations (15 min. for each step instead of 5 min.). 600 ng and 1 µg of final libraries were loaded on two separate PromethION flow cells (R9.4.1 pore version). Base calling was done using albacore 2.3.1.

Altogether, 462 Gb Nanopore reads and 73 Gb PacBio HiFi reads were used to construct the Cameor v2 genome assembly.

### High-throughput chromatin conformation capture (Hi-C)

To produce a high-quality chromosome level assembly, Hi-C maps were produced as described at https://www.dnazoo.org/methods. HiC sequencing was performed at GeT-PlaGe (INRAe Toulouse). Library quality was assessed using an Advanced Analytical Fragment Analyser and libraries were quantified by QPCR using the Kapa Library Quantification Kit. Sequencing has been performed on an Illumina HiSeq. 3000 using a paired-end read length of 2 × 150 pb with the Illumina HiSeq. 3000 Reagent Kits. Altogether, 780 million paired-reads have been sequenced, representing a total length of 218.8 Gb, or 55 X pea genome size. Cis/Trans contact ratios were computed by chromosome on the final assembly using the R library HiContacts version 1.12.0. The mean for all chromosomes was 0.95 which denotes a near equality of cis and trans.

### Optical maps

Optical maps were produced at CNRGV (https://cnrgv.toulouse.inrae.fr/fr) using the Bionano Irys system as described in Aury *et al*.^12^. uHMW DNA was purified from 1 g of dark treated young leaves according to the Bionano Prep Plant tissue DNA Isolation Liquid Nitrogen Grinding Protocol (30177 - Bionano Genomics) with the following specifications and modifications. Briefly, the leaves were fixed in fixation buffer containing formaldehyde, rinsed, manually chopped and then disrupt with rotor stator in homogenization buffer. Nuclei were washed and then embedded in agarose plugs. After overnight proteinase K digestion in the presence of Lysis Buffer and one hour treatment with RNAse A (Qiagen), plugs were washed three times in 1x Wash Buffer and three times in 1x TE Buffer (ThermoFisher Scientific). Then, plugs were melted two minutes at 70 °C and solubilized with 2 µL of 0.5 U/µL AGARase enzyme (ThermoFisher Scientific) for 45 minutes at 43 °C. A dialysis step was performed in 1x TE Buffer (ThermoFisher Scientific) for 45 minutes to purify DNA from any residues. The DNA samples were quantified using the Qubit dsDNA BR Assay (Invitrogen) and the presence of mega base size DNA was visualized using pulsed field gel electrophoresis (PFGE). Labeling and staining of the uHMW DNA were performed according to the Bionano Prep Direct Label and Stain (DLS) protocol (30206 - Bionano Genomics). Briefly, labelling was performed by incubating 750 ng genomic DNA with 1 × DLE-1 Enzyme for 2 hours in the presence of 1 × DL-Green (Bionano Genomics) and 1 × DLE-1 Buffer. Following proteinase K digestion and DL-Green cleanup, the DNA backbone was stained by mixing the labeled DNA with DNA Stain solution (Bionano Genomics) in presence of 1 × Flow Buffer and 1 × DTT (Bionano Genomics), and incubating overnight at room temperature. The DLS DNA concentration was measured with the Qubit dsDNA HS Assay (Invitrogen) and was loaded on the Saphyr chip. Loading of the chip and running of the Bionano Genomics Saphyr System were all performed according to the Saphyr System User Guide(30247 - Bionano Genomics). Data processing was performed using the Bionano Genomics Access software https://bionanogenomics.com/support-page/bionano-access-software/). 1 152 Gb of filtered data (>150 kb) were produced and assembled producing 391 genome maps with a N50 of 25 Mbp for a total genome map length of 3808 Mbp.

### Fluorescence *in situ* hybridization

The mitotic chromosomes used for FISH were prepared from synchronized root tip meristems as described^13^. Satellite repeats were detected either with single oligonucleotide probes or oligonucleotide pools designed according to their consensus sequences and labelled with 5′-biotin during synthesis (Integrated DNA Technologies, Leuven, Belgium). The list of probe sequences is available from [ZENODO repository available at 10.5281/zenodo.17778456]. The FISH experiments were performed as described^8^, with hybridization and wash temperatures adjusted to account for AT/GC content and stringency of hybridization, allowing for 10–20% mismatches. Slides were counterstained with 4’,6-diamidino-2-phenylindole (DAPI) in Vectashield mounting medium (Vector Laboratories, Burlingame, CA, USA) and examined using a Zeiss AxioImager.Z2 microscope with an Axiocam 506 monocamera. Images were captured and processed using ZEN 3.2 software (Carl Zeiss GmbH).

### ChIP-seq detection of centromeric chromatin

The positions of the centromeres in the assembled pseudomolecules were determined by mapping the ChIP-seq reads generated for the two variants of pea CENH3 proteins, CENH3-1 and CENH3-2^9,13^. Raw CENH3 ChIP-seq data are available from the ENA European Nucleotide Archive https://identifiers.org/ena.embl:PRJEB54858 (2023) under accession numbers ERR9981080-ERR9981086. The ChIP-seq experiments were performed with native chromatin^7^ using custom antibodies. DNA fragments isolated from the immunoprecipitated samples were sequenced together with the corresponding control samples (input; digested chromatin not subjected to immunoprecipitation) on the Illumina platform (Admera Health, NJ, USA) in paired-end, 150 bp mode. Duplicate experiments, including independent chromatin preparations, were performed for each CENH3 variant with either one antibody (P23 for CENH3-2 targeting the epitope “TPRHARENQERKKRRNKPGC”) or with two different antibodies (P22 and P43 for CENH3-1 targeting the same epitope “GRVKHFPSPSKPAASDNLGKKKRRCKPGTKC” but raised in rabbit and chicken, respectively).

### Centromeric regions

Due to the large size and repeat complexity of the pea centromeres, these regions were subjected to additional rounds of review and possible correction using the following procedure: (i) the order and orientation of contigs was verified by the presence of ultra-long nanopore reads spanning the gaps between adjacent contigs, (ii) when possible, incomplete arrays of satellite repeats were extended or fully included using contigs generated by assembling HiFi reads as described in Macas *et al*.^9^, (iii) the presence and arrangement of specific satellite repeats (PisTR-B and FabTR-1, FabTR-7, FabTR-9, FabTR-10, FabTR-12, FabTR-85, FabTR-107)^8,9^ was verified by fluorescence *in situ* hybridization on mitotic chromosomes.

The centromere structure was analyzed by combining CENH3 ChIP-seq data with repeat and gene annotations. The ChIP-seq reads were quality filtered and trimmed using Trimmomatic^14^ (minimum allowed length = 100 nt), yielding 122–211 million reads per sample, which were mapped to the assembly using Bowtie 2 version 2.4.2^15^ with the options -p 64 -U. The subsequent analysis was performed with the complete output of the Bowtie 2 program and with the output in which all multimapped reads were filtered out. The filtering of multimapped reads was performed with Sambamba version 0.8.1^16^ with the options “-F [XS] =  = null and not unmapped and not duplicate”. Regions with statistically significant ChIP/input enrichment ratios were identified by comparing ChIP- and input-mapped reads using the program epic2^17^ and the parameter “–bin-size 200”. An alternative identification of enrichment was performed using MACS2^18^ version 2.1.1.20160309 with the default settings. The ChIP/input ratio was calculated for plotting purposes using bamCompare (version 3.5.1) from the deepTools package^19^. The program was run with the parameter “-binSize 200” to calculate the log2 ratio for the window size of 200 nt. The resulting data was recorded using the rtracklayer package from R^20^. The complete Illumina reads preprocessing pipeline and ChIP-seq analysis pipeline were implemented as a Snakemake workflow and executed using a Singularity container. The source code for these pipelines is available at 10.5281/zenodo.14802196 and 10.5281/zenodo.14801796.

**Fig. 1:**
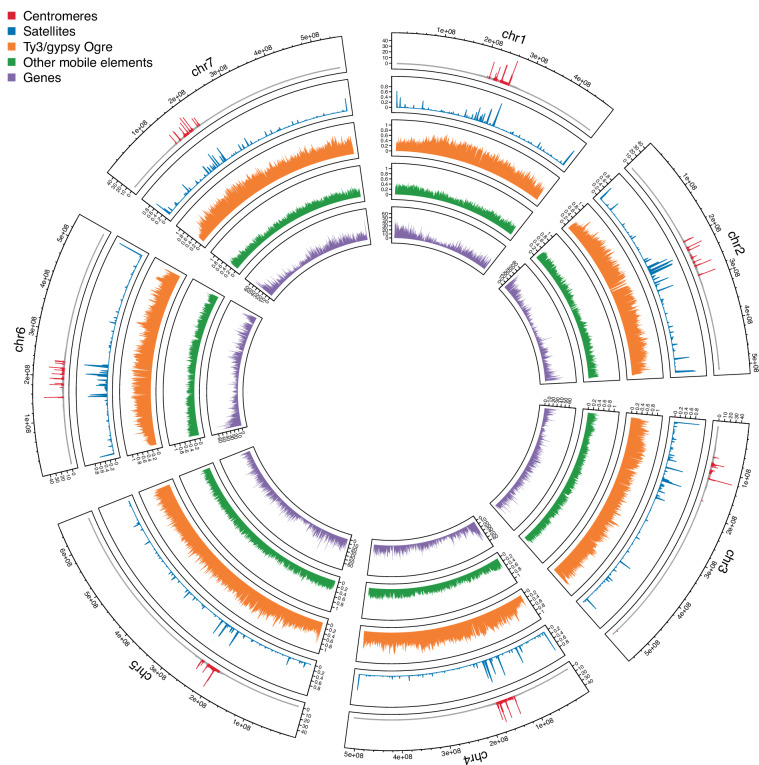
Distribution of genomic features on the chromosomes of *P. sativum* cv. ‘Caméor’. The tracks on the Circos plot are arranged from outside to inside, and represent: (1) position of centromeres as determined by ChIP-seq with the CENH3 antibody, shown as ChIP/input ratio; (2) density of satellite DNA; (3) density of Ty3/gypsy Ogre elements; (4) density of other mobile elements; (5) number of protein-coding genes. All densities, gene numbers and ChIP/input ratios are averaged over 1 Mb windows.

**Fig. 2:**
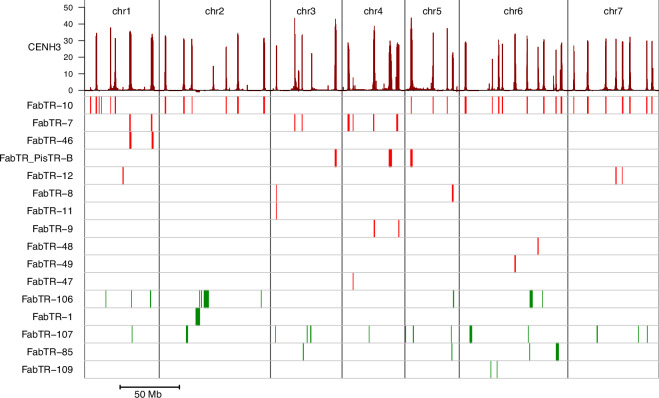
Structure of the centromeres. The upper panel shows the distribution of multiple centromere domains along the centromeric regions (see Table 1 for the coordinates of the plotted regions). The domains are revealed by the increased ChIP/input read ratio in ChIP-seq with the CENH3 antibody. The bottom panel shows the distribution of sixteen families of centromeric repeats, with each row corresponding to a different repeat family. The satellites highlighted in red are associated with CENH3 chromatin.

The centromere positions on the assembled pseudomolecules were defined as regions bounded by the outermost CENH3 domains, which also define the extent of the primary constriction on metaphase chromosomes, and that can extend from 35.8 Mb (CEN5) to 83.9 Mb (CEN2) (Fig. 1). The coordinates of these regions on the seven chromosomes are given in Table 2. The CENH3 chromatin domains were mostly located on the arrays of satDNA and there were eleven different satDNA families associated with CENH3. In addition, there were arrays of CENH3-free satellites in all centromeres. The distribution patterns of satDNA were unique for each centromere (Fig. 2). In-depth analysis of Chip-seq distribution in the genome is possible using the JBrowse browser: http://w3lamc.umbr.cas.cz/lamc/?page_id=8.

**Table 2:** Coordinates of the centromeres and number of telomeric regions detected and their location on the seven chromosomes of Cameor_v2 genome assembly.

Chromosome	Centromere		Telomeres
	Start	End	Number present (position)
1	209389100	262299400	1 (0–30000)
2	205152300	289014100	1(508910000–508942171)
3	71156400	121514600	0
4	136927100	179890900	1 (508040000–508065267)
5	206443100	242222800	1 (656220000–656251770)
6	152399800	234037500	1 (0–20000)
7	137100700	203943200	2 (0–20000; 551830000–551846422)

### Chromosome-scale genome assembly

**Fig. 3:**
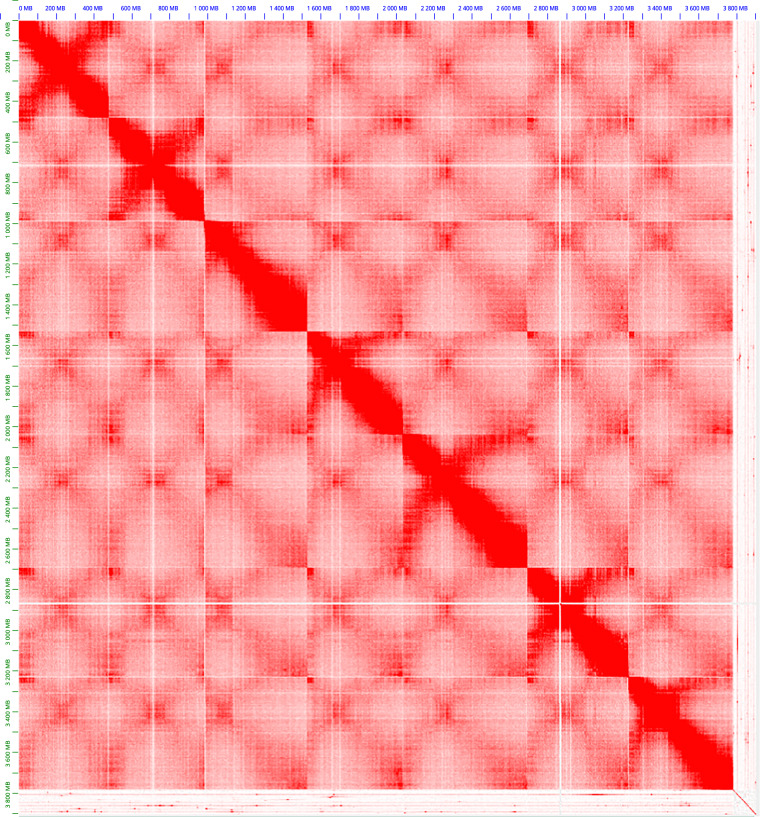
HiC contact map. HiC reads were mapped onto the final Cameor_v2 genome assembly to confirm the high-quality of the assembly.

Nanopore reads were trimmed using porechop^21^ v0.24 by defaults and filtered with NanoFilt^22^ v2.8.0 to keep only those with a quality of 8 and a length of at least 10 kb using parameters -q 8 -l 10000. Contigs were assembled using flye v2.8,1 with parameters --genome-size 4.35 g–asm-coverage 40^23^ and then scaffolded using Hi-C data with the Juicer^24^ v1.6 and 3D-DNA pipeline^25^. The assembly was refined through a round of manual corrections with JuiceBox Assembly Tools^26^. Bionano map was used to detect breakpoints in the assembly, that were subsequently corrected using Bionano solve v3.5.1. Actually, there were few conflicts revealed by the Bionano and genetic maps. Only one scaffold with a size of 8,7 Mb was manually re-oriented using the Bionano, genetic maps and remapping of Hifi reads on chromosome 3 at 154089525 bp from the start. Genetic maps^27,28^ were then anchored using^29^ ALLMAPS v1.1.11 to assess chromosome sequences. The assembled pseudo-molecules were corrected first by two rounds of Racon^30^ using nanopore long reads and one round of medaka v1.3.2 (“GitHub - Nanoporetech/Medaka: Sequence Correction Provided by ONT Research” n.d.). Then, the genome sequence was polished by one round of Polca^31^ with short-reads and one with HiFi long reads. Centromeres were manually corrected^9^ using FISH data, that physically placed probe sequences as described above. Finally, assembly gaps were filled using HiFi sequence reads. To check the quality of the final assembly, Hi-C sequencing data were then remapped on the final genome assembly (Fig. 3) using the arima mapping protocol (https://github.com/ArimaGenomics/mapping_pipeline). Completeness and base-level accuracy were assessed with Merqury^32^. First, k-mers from the reads used for assembly were counted with meryl (same reference) using the command meryl count k = 21. Merqury was then run with default parameters, using the resulting meryl k-mer database and the final assembly FASTA as input. QV scores and completeness values were calculated for both the previous and the current assembly to enable direct comparison.

### Repetitive DNA annotation and masking

Tandem repeats and satellites were annotated using TideCluster v.1.6 (10.5281/zenodo.7885625), a wrapper for TideHunter^33^. Satellite repeats with a monomer size ranging from 40 bp to 3 kb and a minimum array length of 5 kb were annotated using the default TideCluster settings. Satellites with a monomer size between 10 to 39 bp and a minimum array length of 5 kbp were identified using TideCluster with parameters -T “-p 10 -P 39 -c 5 -e 0.25” -m 5000.

LTR retrotransposons (LTR-RT) were annotated using DANTE v0.2.5 and the DANTE_LTR v0.4.0.4 pipeline^34^ on the RepeatExplorer Galaxy server^35^. The sequences of the identified LTR-RT elements were used to create a custom library of LTR-RT elements using the “dante_ltr_to_library” script from the DANTE_LTR repository (10.5281/zenodo.7891007).

A custom library of Class II transposable elements was obtained from previously published datasets^36,37^ and using RepeatExplorer clustering procedure 1^38^ on unassembled Illumina paired-end reads. Contigs corresponding to Class II retrotransposons with a minimum read depth of 5 reads and a minimum length of 100 bp were obtained using tools on the RepeatExplorer Galaxy server. A custom library of LINE elements was created by extracting regions with LINE protein coding domains identified by DANTE, along with the upstream and downstream 4 kb flanking regions. The extracted genomic sequences were split into 100 nt fragments and analyzed by RepeatExplorer clustering. Contigs corresponding to LINE elements with a read depth of at least 3 reads and a minimum length of 150 nt were converted into a custom library. Consensus sequences of rRNA gene arrays including intergenic spacer sequences were fully reconstructed from the RepeatExplorer contigs.

All custom libraries were concatenated and used as a library for RepeatMasker^39^ search. The RepeatMasker (v 4.1.5) search was performed with options “-xsmall -no_is -e ncbi”. All regions annotated as mobile elements with RepeatMasker based on custom library search which overlapped with satellite repeats annotated by TideCluster were removed from the annotation using bedtools (v 2.31.1) with command “bedtools subtract”.

The resulting GFF3 was then merged with the DANTE annotation using a custom R script. The classification of mobile elements in the annotation files corresponds to the classification system used in the REXdb database^40^.

For the final repeat-masking process, all of the above repeat annotation GFF3 files were consolidated. We merged the annotated regions into a single BED file using the bedtools merge tool^41^.

The complete repeat annotation pipeline was implemented as a Snakemake workflow and executed using a Singularity container. The source code for the pipeline is available at 10.5281/zenodo.14801742.

Furthermore, we searched for telomeric repeat sequences using tidk^42^ (v0.2.41) with default parameters and the ‘-clades Fabales’ option. We identified telomeric regions based on the presence of the highly repeated sequence AAACCCT.

### Gene annotation

Several RNA-seq datasets were mapped onto the Cameor v2 assembly using STAR^43^ v2.7.10b with the following options:--outSAMstrandField intronMotif--twopassMode Basic--outFilterMultimapNmax 20--alignSJDBoverhangMin 1. Datasets correspond to different plant tissues produced under control conditions^44^ (PRJNA267198); to developing seed tissues under water and sulfur stress^45^ (PRJNA517587); to plant tissues produced under cold-stress^46^ (PRJNA543764). Only reliable junctions for each alignment files were kept using Portcullis^47^ v1.2.4. Stringtie2^48^ v1.3.6 was then used to assemble a transcriptome for each condition, merging replicates when this was possible. Then, Mikado^49^ v2.3.4 selected the best transcript for each dataset, yielding four different assemblies.

Transcriptomes assemblies and protein sets from different legumes (*V. faba, L. culinaris, M. truncatula, L. japonicus)* were used as hints to run the Eukaryote EuGene pipeline version 1.6.5^50^. Helixer^51^ v0.3.2 was run on the genome using the land_plant 0,3 model and general recommendations for plants (subsequence-length at 64152, overlap-offset at 32076 and overlap-core-length at 48114). Both annotations were then merged using gffcompare^52^ v0.12.6 and functionally annotated using emapper v2.1.9 on eggNOG 5,0^53^. Only gene models with less than 50% of their coding sequences overlapping a transposable element and not functionally annotated as a repeat were kept. Terms used for this functional annotation screening were: transposon|LTR|copia|GAG|helicase|integrase|gypsy|transposition|ribonuclease|transcriptase|polymerases|mobile. The distribution of genes and repeated elements across the genome is represented on Fig. 1.

The Cameor_V2 genome assembly includes 49667 genes, corresponding to 3.3% of the final assembly. The quality of gene annotation was compared with other pea genome assemblies published recently. A BUSCO^54^ analysis using the “fabales” lineage showed that Cameor_v2 has more complete duplicate and slightly less missing genes than other published pea genome assemblies (Table 3), probably thanks to an increased completion of gene detection and sequences. The Cameor v2 assembly contains 83.27% repetitive sequences (Table 4), predominantly LTR retrotransposons (80.27% of the genome), including 13.45% Ty1/Copia and 66.60% Ty3/Gypsy elements. The most abundant LTR retrotransposon lineage is Ogre. Class II elements (DNA transposons) represent only 2.52% of the genome, and satellite repeats account for 2.79% of the assembly.

**Table 3:** Assembly statistics of Cameor_v2 and published pea genome sequences of Cameor_v1^2^, ZW6^3^, ZW1^4^ and JI2822^5^.

Assembly statistics	Cameor_v1	Cameor_v2	Zhongwan6	Zhewan1	JI2822
Total assembly length (Mb)	3,920.16	3,898.56	3,926.41	3,796.70	3,842.02
Total chromosome length (Mb)	3,234.74	3,778.22	3,719.09	3,790.21	3,783.35
Unassigned contigs’ length (Mb)	0.0685	0.1203	0.2073	0.0649	0.05866
*Unassigned contigs/total assembly lengths*	*0.175*	*0.031*	*0.053*	*0.002*	*0.015*
Number of contigs	21,801	3,141	2,402	4,984	2,815
Max size of contigs (Mb)	—	0.0891	0.07263	0.02392	0.02479
N50 contigs (Mb)	0.4159	22.7896	8.9891	4.2300	4.0848
Number of scaffolds	14,274	1,394	1,581	2,906	170
Max size of scaffolds (Mb)	579.27	656.25	652.93	666.97	647.40
N50 scaffolds (Mb)	446.4	537.5	523.4	533.4	535.0
Sum of gap size (Mb)	760.659	0.648	10.242	3.334	1.287
*Ratio of Gaps/total assembly lengths*	*0.194*	*0.0002*	*0.0026*	*0.0009*	*0.0003*
% GC	37.6	37.88	37.84	37.79	37.89
**BUSCO**					
Complete single copies	94.2%	93.1%	93.8%	93,90%	94%
Complete duplicated	3.2%	5.2%	4.4%	4,1%	4,1%
Fragmented	0.6%	0.4%	0.4%	0,5%	0,4%
Missing	2.0%	1.3%	1.4%	1,5%	1,5%
**Gene annotation (without isoforms)**					
Number of genes	44,756	49,667	47,526	—	33,559
Number of mrnas with UTR both sides	18,716	33,500	118	—	5
Number of mRNAs with at least one UTR	22,446	35,280	2,610	—	5
Number of exon	193,289	214,820	218,023	—	146,466
Total gene length (Mb)	124.64	128.27	141.09	—	98.02
Total intron length (Mb)	59.83	54.88	79.66	—	56.50

Contig statistics were obtained from final published assemblies.

**Table 4:** Assembly Repeat Composition.

Repeat type	Total size [Mbp]	Genome Proportion [%]
Class_I	3,136.7	80.46
LINE	5.9	0.15
LTR	3,129.5	80.27
Ty1/Copia	524.5	13.45
SIRE	318.1	8.16
Angela	91.9	2.36
Ivana	52.1	1.34
Ale	28.0	0.72
Other Ty1/Copia	34.3	0.88
Ty3/Gypsy	2,596.6	66.60
Ogre	2,140.0	54.89
Athila	251.6	6.45
Tekay	180.2	4.62
Other Ty1/Gypsy	24.7	0.6
Class_II	98.4	2.52
Low complexity	29.3	0.75
Satellites	108.8	2.79
Unclassified	0.8	0.02
Total	3,246.3	83.27

### Resources for genetics, comparative and functional genomics

RNA-seq datasets used for annotation were mapped onto the Cameor v2 assembly. Moreover, the SNP context sequences of genotyping arrays were mapped onto this genome version: the Infinium GENOPEA array^27^ and the Axiom array^28^. The set of exome capture SNP^55^ was recalled on the genome. Briefly sequenced reads were trimmed for adaptor sequence using cutadapt 1.8.3^56^. The GATK variant calling best practices pipeline was run on the new version exome content using elprep 5.1.3^57^, and GATK 4.2.6.1^58^ was used to merge and produce a SNP VCF file. Furthermore, Cameor_v2 was integrated in the OrthoLegKB database^59^ in order to enable comparative genomics and translational approaches using this new genome version. Orthologs between Cameor_v2 and other legumes’ genomes were detected using Orthofinder v2.5.4 and syntenic relationships were investigated as described in Imbert *et al*.^59^.

Functional annotation was conducted using two different databases. First, TRAPID 2.0^60^ was launched by the web interface using Dicots PLAZA 4.5^61^ to provide by orthology Gene Ontology identifier for 31602 genes. Eggnog-mapper v2.1.12^62^ was launched on EggNOG database 5.0 to provide the human readable annotation that were included in the gene annotation gff for 34543 genes. Other type of annotations, BIN from Mercator4v.6^63^, or provided by emapper, like the Enzyme Classification or the PFAM identifiers were compiled into an R org.DB package using AnnotationForge v1.10.1 (10.18129/B9.bioc.AnnotationForge) to facilitate their access.

## Data Records

All sequencing data generated in this study are available at ENA European Nucleotide Archive https://identifiers.org/ena.embl:PRJEB78861^64^. The chromosome-scale genome assembly has been deposited in the same ENA European Nucleotide Archive https://identifiers.org/ena.embl:PRJEB78861^64^. The genome assembly is also available at https://identifiers.org/insdc.gca:GCA_977071245.1^65^. In addition, the chromosome-scale genome assembly and all associated data have been deposited in the Research data gouv data store 10.57745/5MDE99^66^: the Cameor_v2 genome sequence and annotated gene CDS and proteins, prefixed with CAMEOR_V2, are available in fasta format and the genes and repeats annotation files, prefixed with CAMEOR_V2, are available in gff3 format. This data store also contains an EGGNOG functional annotation file, a file with the correspondence between Cameor_v1 and v2 genes, and published complementary RNAseq and SNP data, that were mapped onto the Cameor_V2 genome sequence. Genomic browser (JBrowse) including assembly annotation and ChIP-seq data tracks can be accessed from http://w3lamc.umbr.cas.cz/lamc/?page_id=8.

## Technical Validation

**Fig. 4:**
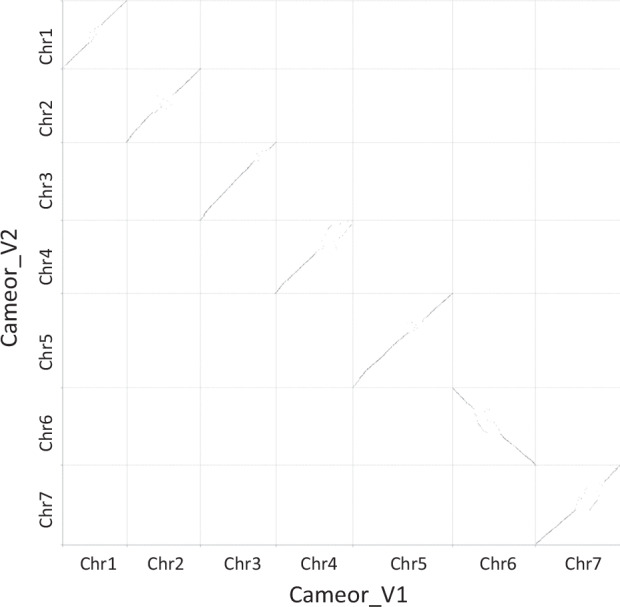
Dot-plot comparing the Cameor_v2 genome sequence with the published pea genome sequence Cameor_v1^2^.

Cameor_v2 final assembly is 3.899 Gb long. Seven scaffolds corresponding to the 7 pea chromosomes represent 96.9% of the total assembly (3.778 Gb) and 1387 unassigned scaffolds represent 3.1% of the total assembly (120 Mb). Out of the 1387 unassigned scaffolds, 92 scaffolds (total length 52 Mb) were found to be enriched in CENH3 ChIP-seq signal. These 92 scaffolds likely represent centromeric regions that could not be confidently placed in the pseudomolecules. Nanopore long-reads, Hi-C scaffolding and a careful manual curation of centromeric regions, led to a significantly more continuous genome assembly, with smaller total gap size (only 648 kb) and a larger contig N50 (22.78 Mb; Table 3). A manual validation of the assembly was done using Bionano maps. Two super-scaffolds were reoriented based on this curation. Additionally, the assembly of centromeric regions was verified by visualization of specific families of satellite DNA using FISH. The HiC contact map confirmed the high quality of the assembly and the repetitive nature of centromeres (Fig. 3). Analysis of the k-mer spectra from the input reads and the final assembly using Merqury showed a completeness of 97.65% and a QV score of 33.75, corresponding to a base-level accuracy of 99.95%. The previous version of the Cameor assembly had a completeness of 95.09% and a QV score of 23.80 (99.58% accuracy). These results demonstrate an improvement in both completeness and base-level accuracy in the current assembly compared with the previous version. The comparison of Cameor_v2 to Cameor_v1 using Chromeister V1.5a confirmed that this genome assembly was significantly improved, especially for centromeric regions (Fig. 4).

Thanks to the refined assembly of centromeres in Cameor_v2, the orientation of chromosomes was revised according to the internationally accepted karyotype rules, with the short arm being placed at the top, and the long arm at the bottom of chromosomes. Chromosome sequences of Cameor_v2 thus start from the telomere of the short arm, through the centromere, to the telomere of the long arm at the end of the pseudomolecule. This led to the inversion of chromosomes 1, 2, 3, 4, 5 and 7 in Cameor_v2 as compared to Cameor_v1 (Fig. 4).

**Fig. 5:**
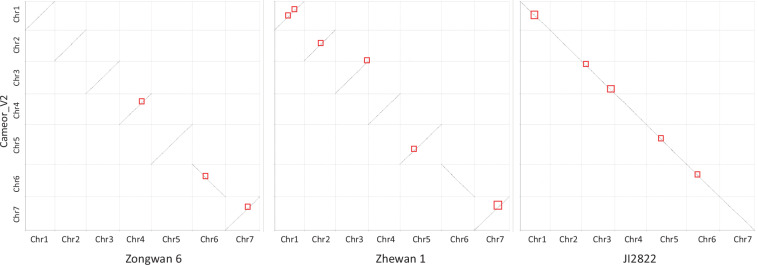
Dot-plot comparing the Cameor_v2 genome sequence with the published pea genome sequences of ZW6^3^, ZW1^4^ and JI2822^5^ using Chromeister V1.5a; red squares highlight breaks in collinearity.

Comparing some published pea genome sequences using Chromeister (Fig. 5) highlighted the high collinearity between Cameor_v2 and genome assemblies of ZW6, ZW1, and JI2822. Some breaks in collinearity occur, mostly near centromeres or telomeres (Fig. 5). Some more complex patterns were identified with ZW1. These breaks in collinearity may be due to real rearrangements (inversions, duplications) in the genomes of ‘Caméor’, ‘Zhongwan 6’ and ‘Zhewan 1’; or may be due to assembly artifacts, in regions that are difficult to assemble due to repetitive elements.

**Fig. 6:**
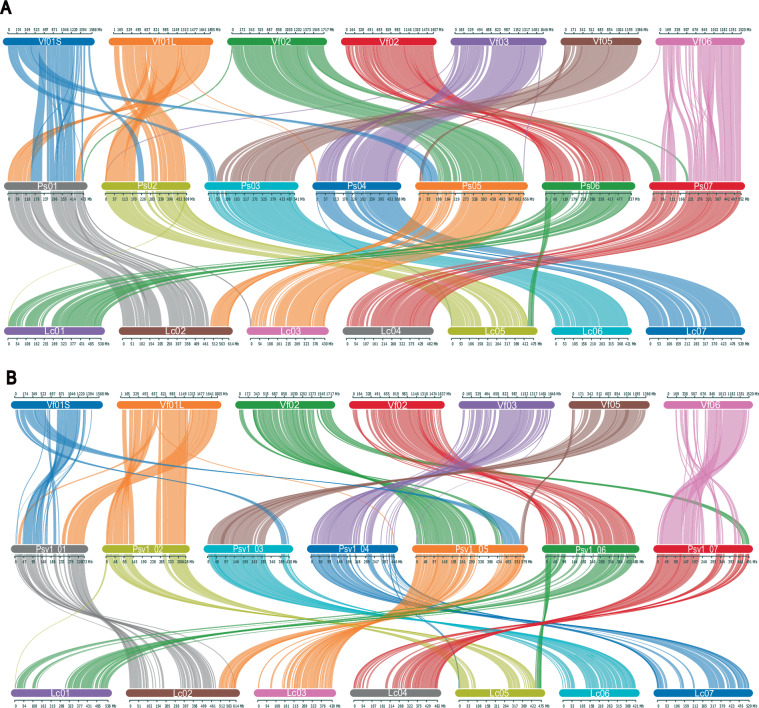
Synteny between chromosomes of *Pisum sativum* (Ps01 to Ps07), *Vicia faba* (Vf01 to Vf06)^63^, *Lens culinaris* (Lc01 to Lc07)^64^. A: using *P. sativum* Cameor_v2 genome; B: using *P. sativum* Cameor_v1 genome. Synteny was visualized using SynVisio^74^.

Assessing synteny and collinearity conservation between Cameor_v2 and other legume genomes (faba bean, lentil, chickpea, and the model species *M. truncatula* Gaertn.) showed that improving the quality of the genome sequence of ‘Caméor’ helped to detect and refine the syntenic relationships with these legumes’ genomes (Fig. 6). Larger syntenic blocks were identified for all chromosomes and gaps in centromeric regions were reduced in length (Fig. 6). Syntenic blocks, i.e. conserved chains of orthologous genes across chromosome pairs, were fewer, but longer and included more genes when Cameor_v2 was used, as compared to Cameor_v1, certainly due to improved gene annotation and gene order (Table 5).

**Table 5:** Statistics of syntenic relationships between the pea genome assemblies Cameor_v1 and Cameor_v2 and genome assemblies from faba bean^67^, lentil^68^, chickpea^69^, and *M. truncatula* Gaertn^70^.

Statistics	Cameor_v2	Cameor_v1
Total number of protein-coding genes	49,667	44,958
Number of genes with orthologs	33,072	30,439
Number of orthologous collinear genes	20,457	12,771
% of collinear genes among orthologs	62	42
Mean orthologous gene count per block	96	46
Number of syntenic blocks	703	814
Mean block size	18,471,838	11,063,388

Only orthologous genes found in the Cameor genome were considered. OrthoFinder^71^ was employed to identify orthogroups, using the genome of mung bean (*Vigna radiata* L.)^72^ as an outgroup. Orthogroups served as anchors to define syntenic blocks among the genome sequences, using MCScanx^73^. MCScanX was run with its default parameters, except for the minimum number of genes required to define syntenic blocks, which was set to 10.

## Data Availability

All sequencing data generated in this study and the chromosome scale genome assembly are available at ENA European Nucleotide Archive https://identifiers.org/ena.embl:PRJEB7886165. The genome assembly is also available at https://identifiers.org/insdc.gca:GCA_977071245.166 and in the Research data gouv data store at 10.57745/5MDE99^66^.

## Code availability

Repeat Annotation: 10.5281/zenodo.14801742. Illumina preprocessing: 10.5281/zenodo.14802196. ChiP-Seq analysis: 10.5281/zenodo.14801796.
